# Webcam eye tracking close to laboratory standards: Comparing a new webcam-based system and the EyeLink 1000

**DOI:** 10.3758/s13428-023-02237-8

**Published:** 2023-10-11

**Authors:** Tobiasz Kaduk, Caspar Goeke, Holger Finger, Peter König

**Affiliations:** 1 Institute of Cognitive Science, University of Osnabrück, Osnabrück, Germany; 2 Research and Development Division, Scicovery GmbH, Paderborn, Germany; 3 Department of Neurophysiology and Pathophysiology, University Medical Center Hamburg-Eppendorf, Hamburg, Germany

**Keywords:** Labvanced, Eyetracking comparison, Online testing, Webcam based eyetracker

## Abstract

This paper aims to compare a new webcam-based eye-tracking system, integrated into the Labvanced platform for online experiments, to a “gold standard” lab-based eye tracker (EyeLink 1000 - SR Research). Specifically, we simultaneously recorded data with both eye trackers in five different tasks, analyzing their real-time performance. These tasks were a subset of a standardized test battery for eye trackers, including a Large Grid task, Smooth Pursuit eye movements, viewing natural images, and two Head Movements tasks (roll, yaw). The results show that the webcam-based system achieved an overall accuracy of 1.4°, and a precision of 1.1° (standard deviation (SD) across subjects), an error of about 0.5° larger than the EyeLink system. Interestingly, both accuracy (1.3°) and precision (0.9°) were slightly better for centrally presented targets, the region of interest in many psychophysical experiments. Remarkably, the correlation of raw gaze samples between the EyeLink and webcam-based was at about 90% for the Large Grid task and about 80% for Free View and Smooth Pursuit. Overall, these results put the performance of the webcam-based system roughly on par with mobile eye-tracking devices (Ehinger et al. *PeerJ*, *7*, e7086, 2019; Tonsen et al., 2020) and demonstrate substantial improvement compared to existing webcam eye-tracking solutions (Papoutsaki et al., 2017).

## Introduction

Tracking eye movements provides a comprehensive opportunity to study the allocation of visual attention. Therefore, in areas such as psychology (Rahal & Fiedler, 2019), marketing (Białowąs & Szyszka, 2019), and clinical applications (Harezlak & Kasprowski, 2018), this unique tool is very popular (Kowler, 2011). Fixations, gaze position, saccadic movements, pursuit movements, pupil diameter, and blinks are the key phenomena observed using eye tracking (Holmqvist et al., 2011). Based on the fixation duration, for example, researchers are then able to locate visual elements in psychophysical experiments that caught the subject's attention (Borys & Plechawska-Wójcik, 2017)*.* Further, in the field of neuromarketing, it is common practice to combine gaze data with pupil dilation to study consumer preferences that potentially could reveal human emotions towards certain products or brands (Ungureanu et al., 2017). Analyzing fixations and gaze position is also essential for clinical studies that aim to understand how attention influences the decision-making process and the relative saliency of different objects and related disorders. For instance, Wang et al. (2015) compared eye movements among subjects with autism spectrum disorder (ASD) to a control group. The results suggest that participants with ASD had a stronger image center bias and reduced saliency for faces and locations indicated by social gazes. Given the vast number of possible applications and use cases, eye-tracking technologies and technical development have recently gained popularity (Yang & Krajbich, 2021).

The last two decades have brought substantial advances in eye-tracking technology. Many research laboratories have switched to remote (without chin rest) systems such as the EyeLink System (Bohme et al., 2006). It provides higher comfort for the subjects and similar or even higher accuracy of the system. However, such equipment requires a specialist to operate and the appropriate software, which is characterized by high costs and a low degree of mobility in terms of where the experiment can be performed. Hence, other companies developing eye-tracking technology have focused mainly on mobility and ease of wearing the devices (Niehorster et al., 2020a, b; Tonsen et al., 2020). Modern mobile eye-tracking devices, which often resemble standard glasses, are especially valuable for extended recording sessions, e.g., while driving a car, or for tasks that require strong head or body movements, e.g., during sports activities. While these devices can frequently be worn without expert supervision, they are still relatively expensive. They, hence, are not suited to perform eye tracking for many subjects without enormous costs. One way to reduce costs and drastically increase the availability of eye-tracking research and applications is to use widely available consumer-grade hardware, particularly web cameras and browser-based applications for eye tracking.

Webcam-based eye-tracking technology allows conducting experiments outside the laboratory walls, e.g., at the participant's home. It uses commonly available devices such as personal computers, laptops, mobile phones, or tablets, which are nowadays available in most households (Dupuis & Tsotsos, 2018). Additionally, the use of webcam-based technology does not require the presence of a specialist to conduct the measurements, since the whole procedure is automated and basic skills of operating a laptop, browser, and Internet access are sufficient. In other words, using the webcam for eye tracking introduces the technology to everyone owning and using a computing device with such a camera (Miller & Sinanan, 2014). In line with this, browser-based research has become popular in recent years (Finger et al., 2017a, b). One primary framework that includes webcam-based eye tracking in the browser is called Webgazer, which was introduced in 2016 (Saxena et al., 2022; Papoutsaki, 2015). It marks an important milestone in realizing webcam-based eye tracking as a whole. However, the authors reported an average accuracy of more than 4°, which limits its use for most eye-tracking research. To our knowledge, a significant improvement in accuracy, relative to Webgazer, has not yet been reported for webcam-based eye tracking, especially for a system that works in conjunction with browser-based behavioral experiments. However, given the enormous potential of delivering eye tracking to the general public, further technological development of webcam-based eye-tracking systems is an important area of ongoing research (Semmelmann & Weigelt, 2018). In line with this, the authors of this manuscript developed a new, deep-learning-based architecture for eye tracking that natively works in the browser, and integrated this into the Labvanced platform for online behavioral experiments (Finger et al., 2017a, b). As this webcam-based algorithm has already been accessible for a while in the online platform, a handful of publications utilizing this method have already been published (Sauter et al., 2022; Bánki et al., 2022). However, as a comprehensive test of the algorithm is still missing, we now conducted a systematic comparison study to assess the system's quality.

Evaluating a new eye-tracking system requires considering and testing multiple factors. The subject of privacy-preserving eye-tracking techniques has not been fully addressed in the case of webcam-based eye tracking. The obvious issue here is that the webcam records an RGB video of the participants' faces, which makes it particularly easy to identify subjects, both for human observers and algorithms. When such a video/image of the participants' faces is transmitted to and saved on a third-party server, additional avenues for attack are offered. Therefore, the optimal way to secure data privacy (for webcam-based approaches) by design is to process the image data on the participant's device directly, not to transmit video data and not to store them on a remote server. Thus, it complies with EU regulations: “Personal data shall be: … adequate, relevant and limited to what is necessary for relation to the purposes for which they are processed (‘data minimization’);” (GDPR, Art. 5.1 (c), https://gdpr-info.eu/art-5-gdpr/). Taken together, for evaluating the quality of the proposed webcam-based eye-tracking system, we considered accuracy, precision, and data loss in a variety of tasks including Head Movements, and data privacy concerns as the most critical factors.

Furthermore, it is important to test this functionality of an eye-tracking system for a large variety of tasks. For instance, testing for accuracy and precision requires the subject to fixate on a known target (Large Grid). However, in other tasks, a comparison with ground truth, i.e., the required fixation location, might not be possible if the subjects have no particular task (Free View) (Xia & Quan, 2020; Martin et al., 2020) or are following a moving object (Smooth Pursuit) (Barnes, 2008). In that case, we assess the quality of eye tracking by measuring the correlation of eye-tracking systems. Further, we report data loss and blinks (Head Movements) as important factors affecting eye-tracker data quality, specifically in a setup outside of the laboratory with limited supervision (Franchak et al., 2011). This is because, during the experiment, a subject can change the position of the head to a significant degree so that the system loses the ability to detect the pupil, which leads to data loss. Solutions such as head-mounted, tower chin rest, or remote setup have been proposed to solve this problem and have since significantly improved data quality (Bohme et al., 2006). As online eye tracking is fully automated and typically no specialist is physically present during the experiment for assistance, the method is particularly vulnerable to a drop in accuracy, precision, and an increase in data loss due to head movements without further measures. Accordingly, the accuracy of systems such as Webgazer drops even further when subjects move their heads. These types of limitations and problems are an inseparable element of eye-tracking research.

This publication primarily focuses on the real-time comparison and assessment of the native algorithms of both eye-tracking systems. To compare the classification approaches of the two eye-tracking systems, we utilized the EyeLink algorithm for real-time event detection and a webcam-based dispersion fixation detection algorithm. We decided to employ the built-in algorithms of the two eye-tracking systems in their default off-the-shelf setup. This decision was motivated by the proprietary and closed-source nature of the algorithms used in systems like EyeLink, as well as the limitations of online technologies imposed by the low sampling rate, which change some existing approaches, such as velocity-based algorithms to be impractical. We want to emphasize that our primary focus is on the real-time comparison of the native algorithms in the eye-tracking systems. Additionally, we acknowledge the availability of alternative offline algorithms like I2MC for fixation detection but emphasize that the current publication does not explore offline analysis due to potential variations in challenges, applications, and use cases.

Here, we introduce and test a new webcam-based eye-tracking system integrated into an existing web-based platform to create online experiments (Finger et al., 2017a, b). The authors also implemented and tested head tracking with a virtual chinrest feature (explained in detail in the section below). Functionality relies on real-time head position calculations to mitigate this problem. To evaluate the system, we implemented an established an eye-tracking test battery (Ehinger et al., 2019) and followed the procedures as laid out in this article as far as possible. We recorded eye movements simultaneously with the webcam-based system and a high-end laboratory eye-tracking system (EyeLink 1000 - SR Research). First, the test battery includes a fixation-grid task, which enabled us to calculate the accuracy and precision of both systems. The second task is a Smooth Pursuit, i.e., following a moving object on the screen (Barnes, 2008). Third, a Free View task investigates visual saliency and compares the performance of the two eye-tracking systems' during more natural viewing behavior. Finally, the test battery includes a dedicated test to evaluate the amount of data loss caused by head movements for both systems. Overall, this multidimensional comparison allowed us to answer the question of how the new webcam-based system holds up compared to the EyeLink system, one of the most accurate eye-tracking systems available on the market and often considered the “gold standard” for eye-tracking research. In line with this, we hypothesized that the EyeLink system outperforms the new webcam-based approach by far in pretty much all relevant metrics. That said, we are interested in whether our webcam-based system constitutes an improvement compared to earlier webcam-based approaches. Most importantly, we seek to answer whether the system presented is accurate enough to meet the standards of the research being conducted.

## Methods

### Setup

**Fig. 1:**
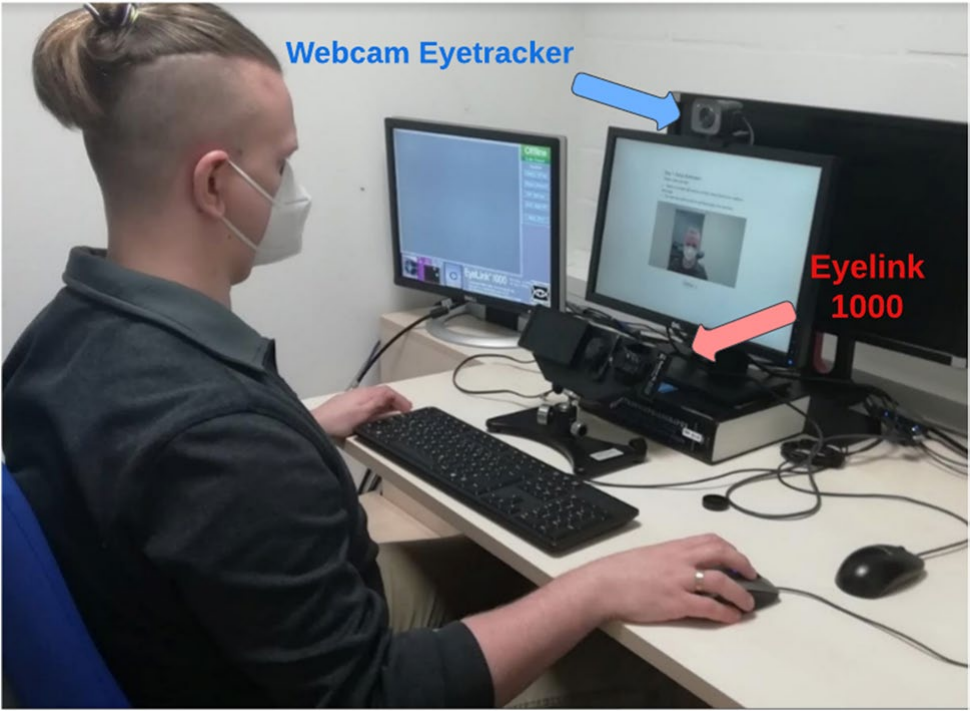
Presentation the entire setup used in the experiment, including EyeLink 1000 and Logitech Webcam. The participants viewed a 15′′ monitor with 1440 × 900 pixels resolution and a 120-Hz refresh rate at a distance of 600 mm. The EyeLink 1000 eye tracker (SR-Research Ltd, Mississauga, Ontario, Canada) was placed directly below the monitor. The webcam was placed on top of the screen. The EyeLink system used a remote mode, i.e., with the remote camera option, with a 500-Hz sampling rate. For the webcam, we used a Logitech StreamCam - Full HD 1080p Streaming Webcam with a maximum sampling frequency of 60 Hz. Even though the Labvanced platform could record data at 60 Hz, for the purpose of this experiment, we set up a sampling rate of 30 Hz to match the majority of webcams on the market at this time. The effective sampling rate observed during the experiment using a webcam-based eye tracker was 29.96 Hz on average

The system was set up such that eye-tracking data could be obtained simultaneously for the EyeLink system and the webcam-based system. During the study, the participants were seated in a room separated from the experimenter (Fig. 1).

The setup included two computers, one for the EyeLink system, called the Host PC, and one for displaying the experiment and calibration, called the Display PC. The experiment was built with the Labvanced Task Editor (Finger et al., 2017a, b) and ran online in the Google Chrome browser. The webcam eye tracking functionality was integrated and controlled by the Labvanced system.

### Synchronization of eye trackers

Synchronization of the two eye trackers was handled via MATLAB (R2016b; MathWorks, Natick, MA, USA) code and Python 3. We sent the UNIX timestamp triggers, which correspond to the start and end of the experiment, from the Display PC to the Host PC. This information was forwarded to the EyeLink data file. For some participants, we experienced a time drift by the difference in the PC's clocks. This was solved using a data-driven approach to time-lag cross-correlation using the Pandas (Python) library built-in function. This in effect eliminates the lag effect between two data streams. After that, data from EyeLink were downsampled and interpolated to the webcam sampling rate standards. The result was one data file where each sample from the eye tracker was matched based on time with the sample from another eye tracker, which made it possible to compare the two systems.

### Participants

The study took place at the Institute of Cognitive Science at Osnabrück University. In total, we recruited 23 participants. Eligibility criteria were set up as follows: no photosensitive migraine or epilepsy, no motion sickness, no drug use, and no glasses. Wearing glasses during eye tracking can lead to additional light reflections, which can negatively affect data quality. Therefore, it is generally discouraged to wear glasses during the recording session. Since other studies that compared the two types of eye tracking have excluded the use of glasses (Ehinger et al., 2019), we have adopted this procedure as well. Nevertheless, in both systems, recording can be carried out with or without glasses.

Four of the participants were excluded from the analysis. In three of these participants, the cross-correlation procedure used to synchronize the different devices failed, resulting in unreliable data. Please note that the synchronization of the two eye trackers is only required for the particular comparison being conducted. Therefore, it is not a mandatory step when using either of the devices independently. The last participant was excluded due to an insufficient number of detected offset-related fixations (described in the Large Grid task section), which was less than 20% of the overall target number. Interestingly, all of these participants exhibited high initial accuracy scores during calibration for both eye-tracking systems, emphasizing the significance of inspecting calibration data to maintain data quality. Out of the total data gathered from 23 participants, we analyzed the data of 19 participants in the first session. This session included the tasks Large Grid, Smooth Pursuit, and Free View. In the second session, we analyzed data from 17 participants doing Head Movements tasks, specifically Roll and Yaw movements. We compensated the participants with either 9.60€ or 1 credit per hour. The participants gave written consent, and the ethics committee approved the study of Osnabrück University (number 58/2021, issued on December 15, 2021).

### Head tracking

**Fig. 2:**
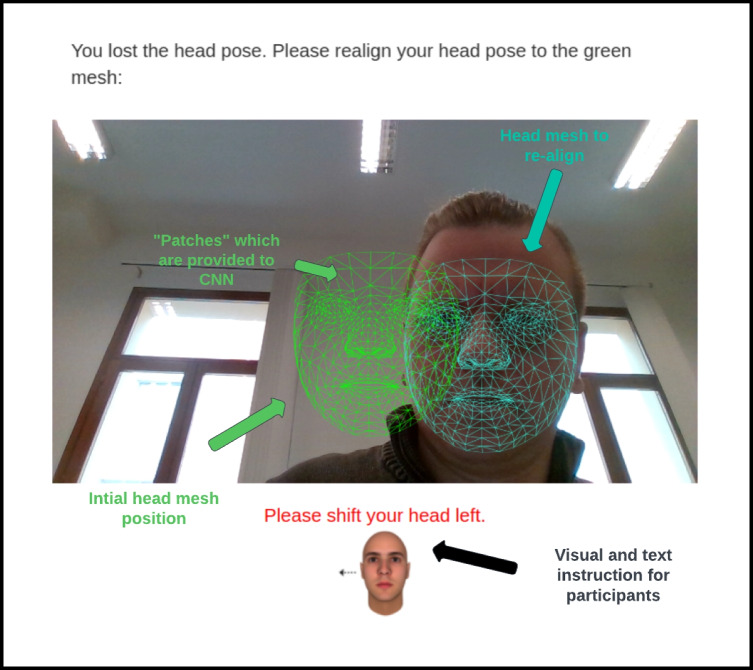
Photo showing the instructions provided to the user. The user follows the instructions described below for the head photo until the blue face mesh is aligned with the green face mesh. When the re-alignment is matched, then the study continues

Head movements are an essential factor negatively affecting eye-tracking data quality (Franchak et al., 2011). The algorithm used for the head-tracking feature is a deep neural network. The algorithm uses a convolutional neural network (CNN) to process the video feed from the participant's webcam in real-time. The CNN works by breaking down the video feed into a series of smaller images, or "patches," (Fig. 2), which are each analyzed independently. The patches are then analyzed by a series of layers within the neural networks, which are designed to identify and classify different features within the image. In the case of the Head Tracking feature, the algorithm is specifically trained to identify and track the participant's face. The neural network is trained using a large dataset of images that includes different head orientations, lighting conditions, and camera angles. By analyzing this dataset, the algorithm can learn to identify the unique features of a human face and track its movement within the video feed.

Once the algorithm has identified the participant's face, it uses a series of mathematical operations to estimate the position and orientation of the head. This information is then used to track the movement of the head and ensure that the participant's eyes remain within the designated area of interest**.**

### Virtual chinrest

Labvanced offers a virtual chin rest that simulates the physical chin rest commonly used in experimental settings to stabilize head movements during tasks. At the start of the study, the subject is allowed to select a comfortable head position. Throughout the study, the subject's head movements are continuously monitored, and the trial is automatically interrupted if the subject moves away from the initial head position. Unlike a physical chin rest, the virtual version provides on-screen feedback and instructions (Fig. 2). The virtual chin rest sensitivity can be adjusted on a six-step scale, ranging from very loose to very strict. This feature is particularly relevant when working with subjects whose head movements are unavoidable or when conducting long-duration studies. For our research, we used the medium-strict sensitivity level to minimize head movements.

### Calibration

Specifically for the comparison, we performed the calibration of two eye trackers one after the other. We used the EyeLink monocular recordings (500 Hz, head free-to-move mode) setup (Ehinger et al., 2019) with nine fixation points on the grid. The researcher's task is to click the *accept* button if the subject properly aims their eyes at the displayed target. This procedure was followed by a validation in which the researcher performed the same procedure. The result given by the EyeLink system was the calibration error average and maximum for each point on the grid. We repeated the calibration procedure until achieving a suitable score (average error < 1.0°, maximum error < 1.5°). EyeLink informs about the acceptable standards via the message “GOOD” (green background, cf. (EyeLink manual) during the calibration process. We chose these calibration criteria because they were introduced in the previous paper comparing EyeLink with the Pupil Lab eye-tracking system (Ehinger et al., 2019).

In our study of webcam-based eye tracking, we adapted the calibration procedure to fit the specific needs of webcam-based eye tracking. This was necessary to account for the differences in camera position and head movements that occur in a non-stationary setup. The Labvanced platform provides users with the option to select from a range of calibration procedures based on the duration time and different subsets of calibration. The calibration procedures offered include a 1-min option, which is the shortest in duration and least accurate, a 5-min option (default), which is a middle solution, and an 8-min option, which is the longest in duration but offers the highest accuracy. For the calibration in this experiment, we chose a default 5-min procedure that involves seven head poses with approximately 12 targets each. The calibration process involves a fixation grid pattern, as well as a brief Smooth Pursuit task and Head Movements task to calibrate the webcam. During calibration, participants are instructed to make slight adjustments to their head position to account for small variations in head position (Fig. 2). Only subjects with a calibration error of less than 7% of the total screen size were allowed to continue in the experiment. In case of a failure, the subject was informed that the calibration accuracy was insufficient and the calibration had to be repeated. This calibration process is designed to meet the highest scientific standards. The official documentation shows a detailed overview of the webcam calibration (https://www.labvanced.com/content/learn/guide/eyetracking/).

### Re-Calibration

Slight changes in lighting, subject fatigue, and other environmental factors can affect data quality during the experiment (Ehinger et al., 2019). Hence, an essential step to maintaining high data quality throughout a study, especially for a webcam-based system, is a re-calibration/drift correction measure. Hence, we choose to recalibrate seven points after every six trials in the Large Grid, every trial for the Smooth Pursuit Task, and every seven trials for Free View. This drift correction feature calculated the cross-validated systematic error across those seven points and subsequently accounted for this in future gaze predictions. Besides such real-time drift correction of the gaze prediction, the re-calibration error can also be saved separately, serving as a continuous measure of measurement reliability. To ensure that the experiment's length will not be disproportionately increased by re-calibration, this procedure is set up to take around 15 s. The overall duration of the re-calibration for the whole experiment should introduce an additional 4 min to the total time of the experiment. Further, in the case of need the re-calibration could be entirely switched off, or the amount of grid points or initialization per trial is customizable within the Trial Editor. This allows us to adjust re-calibration for the longest large-scale studies if this is needed. This type of online eye-tracking calibration using the Labvanced system was successfully implemented in other projects, including infant studies (Bánki et al., 2022). This procedure allows the calibration to be user-friendly and strikes a balance between data quality and the feasibility of experimental procedures.

### Task sequence

**Fig. 3:**
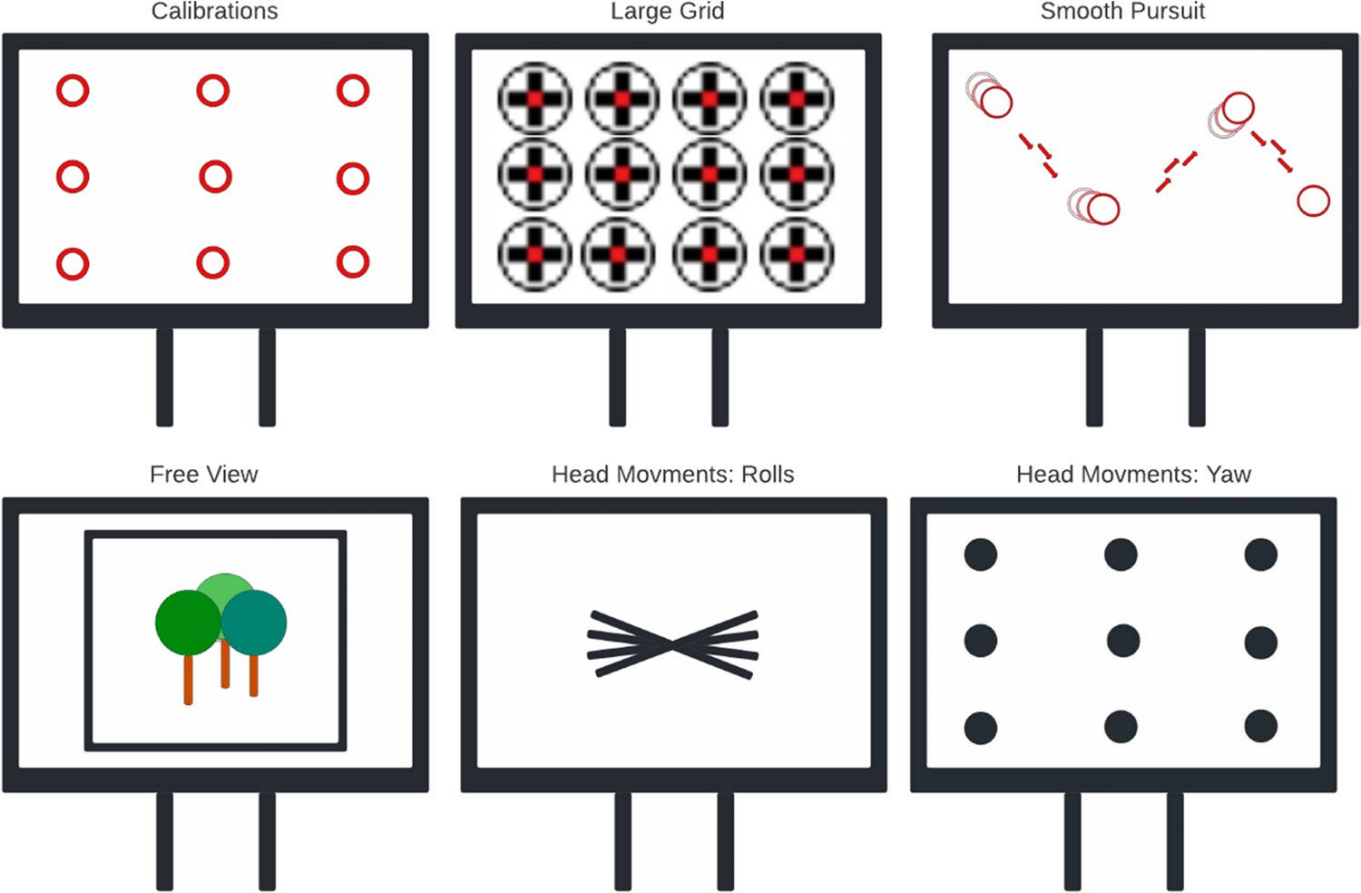
The task sequence used from the top left to the lower right

The battery of tests used for this study was transferred and adapted to online testing conditions from a paper that earlier compared EyeLink with Pupil Labs (Ehinger et al., 2019). For our study, we adopted tasks such as Large Grid, Smooth Pursuit, and Free View. To be able to investigate data loss, we adopted tasks such as Rolls and Yaw Head Movements (Fig. 3).

In the first task, which was Large Grid, we investigated accuracy, precision in the offset-related fixations, gaze data correlation, and data loss. In the Smooth Pursuit and Free View tasks, we were mainly interested in the gaze data correlation, as it is an unbiased measurement that was not processed by any event detection algorithm.

Tasks such as Blink task, Micro Saccades task, and Pupil Dilation task were excluded from the online adaptation for a number of methodological reasons. For example, detecting changes in pupil size may not be feasible with the current webcam hardware standards, as most cameras lack the necessary infrared detection technology to accurately track pupil dilation. Also, detecting micro-saccadic movements was not yet implemented in the webcam-based eye tracker when this experiment was conducted. The Blink task, on the other hand, was moved and merged into the Head Movements task.

### Large Grid task

**Fig. 4:**
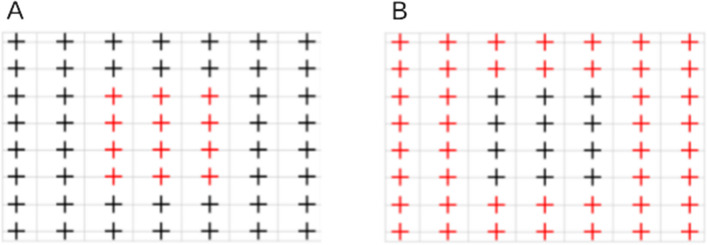
The *red color* marks the targets that were classified as inner in **A** and outer in **B**. We referred to the inner and outer terms further in the analysis section

In the Large Grid task, we presented 56 targets (1.1° black fixation cross with a surrounding circle and red dot in the center) at equidistant intervals in an 8 × 7 grid across the screen. In each trial, we presented one target at a different randomized location. The subject's task was to focus on the presented target that gradually shrank in diameter until it completely disappeared. As soon as the target disappeared, the subject had to press the Spacebar key. We recorded the reaction time of the button press, the timestamp of the target disappearance, and the timestamp of the start and end of fixations directly in the system. We wanted to compare fixations in the most precise way possible. Therefore, we considered only trials in which the subject fixated on the target. We included fixations in the analysis if the button was pressed during the duration of the fixation and if the duration of the button press was below 500 ms. If any of the two criteria were not met, the fixation was not included in the analysis. These stringent criteria were motivated by the fact that we were only interested in the fixations correlated with the offset of displayed stimuli. Therefore, we assessed the criteria individually for each eye tracker, i.e., if a fixation did not meet the criteria for one system, we did not necessarily disregard that fixation when analyzing the data for the other system. The analysis revealed that 58.17% of offset-related fixations were detected by EyeLink, while the webcam-based eye tracker identified 57.80% of such fixations. To determine the accuracy, we calculated the Euclidean distance between the centroid of the selected fixation and the target location of the stimulus. The analysis was conducted separately for inner and outer targets (Fig. 4).

### Smooth Pursuit task

In the Smooth Pursuit task, a red circle was used as a target with a size of 0.52°. The target moved continuously in various directions and speeds while bouncing off the screen edges, and the subject was instructed to follow it. A Wiener process or Brownian motion was applied to the target's movement to create realistic changes in its direction. The variance of the Brownian motion of the direction angle was 887 deg^2^/s. Consequently, the target moved across the screen at different speeds and directions, making it difficult for the subject to predict its acceleration or movement. The experiment is publicly available to inspect and share the template, under this link (https://www.labvanced.com/page/library/28983).^1^

We correlated horizontal coordinates from one eye tracker with horizontal coordinates obtained by another eye tracker. Similarly, the vertical coordinates obtained by one eye tracker are correlated with the vertical coordinates obtained by another eye tracker. This way, we could validate the correctness of the webcam system in terms of raw data output and also were able to distinguish any difference between horizontal and vertical correctness.

### Free View task

In the free-viewing task, photos of natural images with a size of 900 × 720 pixels were presented. Each image was shown for 6 s, and in total, 18 images were shown to each subject. The participants were instructed to explore the images freely, and the 7-point re-calibration was performed after every 7th trial.

For the analysis, we performed several examinations. First, we calculated the correlation of horizontal and vertical coordinates identically as in the Smooth Pursuit task. Second, we calculated and visualized the kernel-density estimate using a Gaussian kernel across all trials and subjects. Third, we selected trials and created a scatter plot for single-trial data, giving a direct visual impression of how similar recorded data were between both systems.

### Head Movements

The Head Movements part of the study consisted of two separate tasks, head roll movements and head yaw movements. In the head roll task, a line was displayed in the center of the screen. The line was then rotated by various degrees (– 15°, – 10°, – 5°, 0°, 5°, 10°, 15°) relative to a horizontal orientation. The subject had to focus the gaze on the line while rolling the head according to the slope of the line. Once this was achieved, the participant had to press the spacebar to confirm the head position. In the yaw task, targets were arranged in a 3 × 5 grid and displayed in a randomized order with one target per trial. The participants were instructed to rotate their heads while they were fixating on the target. Once the subject fixated on the target, they were instructed to press the spacebar to confirm that they assumed the required head position.

For the analysis of both tasks we were interested in how much these movements affected the ability of both eye tracking systems to produce reliable data. Hence, for the EyeLink system, we used the built-in event detection algorithm to calculate the data loss. In the webcam-based approach, we used the confidence measure metric, which was defined as a continuous range between 0 and 1. In our study, data points near 0 were deemed unreliable, while those near 1 were considered reliable. We treated all data marked as 0 as data loss.

### Fixation detection algorithm

**Fig. 5:**
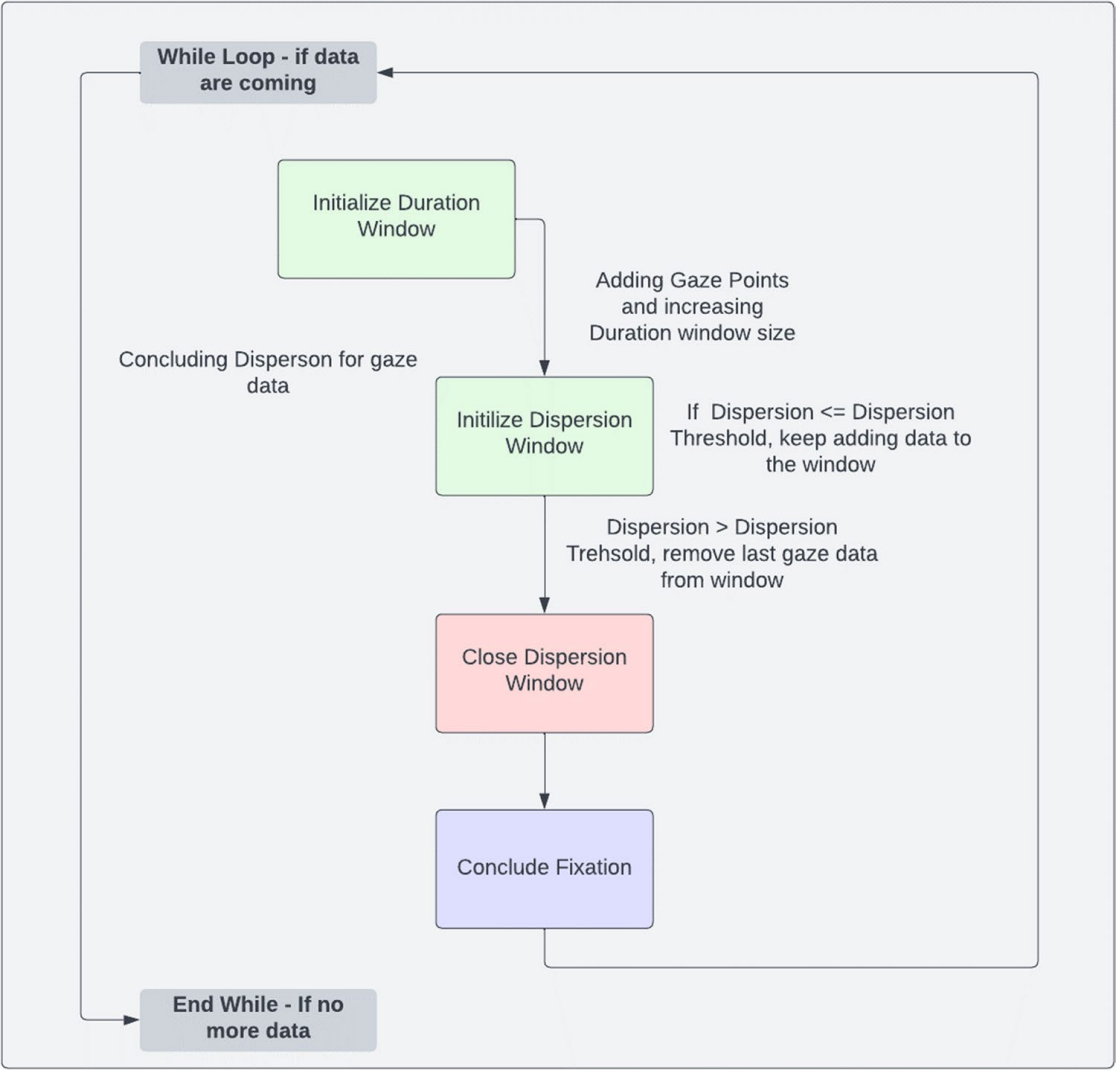
Main concepts of the developed dispersion-based fixation-detection algorithm. First, a new fixation is created if three or more gaze points are found with low enough dispersion. Then more gaze points are added to fixation if both the overall dispersion and also relative dispersion change are below a threshold. It can also lower the dispersion by adding a new gaze point and removing the oldest one. Once a new gaze point exceeds the threshold for dispersion, the fixation is concluded

Due to the unavailability of the fixation detection script from the EyeLink system to the general public, we opted to conduct real-time comparisons of embedded classification algorithms. Among the numerous algorithms available for classifying fixations (Blignaut, 2009), we chose to implement the dispersion-based algorithm (as shown in Fig. 5). A comprehensive description of the algorithm is accessible via the following hyperlink on GitHub, which is open to the public. (https://github.com/Labvanced/eye-tracking-fixation-detection.).

## Results

### Calibration error

Comprehensive information regarding the calibration error of both eye trackers is presented in the following section. For the EyeLink eye tracker, the mean calibration error across all participants was M = 0.50, with a maximum calibration error of M = 1.04. In contrast, for the webcam-based eye tracker, the mean calibration error was M = 1.44 for all participants. It should be noted that the maximum calibration error was not ascertainable for the webcam-based eye tracker Table 1.

**Table 1:** Detailed calibration output from both eye trackers for each subject

	EyeLink calibration output in [°]		Webcam-based eye tracker calibration output in [°]
Participant number	Mean calibration error	Max calibration error	Mean calibration error
1	0.64	1.45	1.38
2	0.35	0.55	0.96
3	0.44	1.13	1.44
4	0.61	0.93	1.37
5	0.39	0.85	1.21
6	0.58	1.52	1.74
7	0.45	0.90	1.09
8	0.59	0.86	1.17
9	0.50	1.38	1.46
10	0.48	1.42	1.59
11	0.42	1.10	1.92
12	0.49	0.95	0.97
13	0.46	0.97	1.77
14	0.55	0.95	1.31
15	0.47	0.67	2.60
16	0.51	0.98	1.20
17	0.63	1.24	1.33
18	0.52	1.28	1.10
19	0.50	0.80	1.76

### Large Grid

**Fig. 6:**
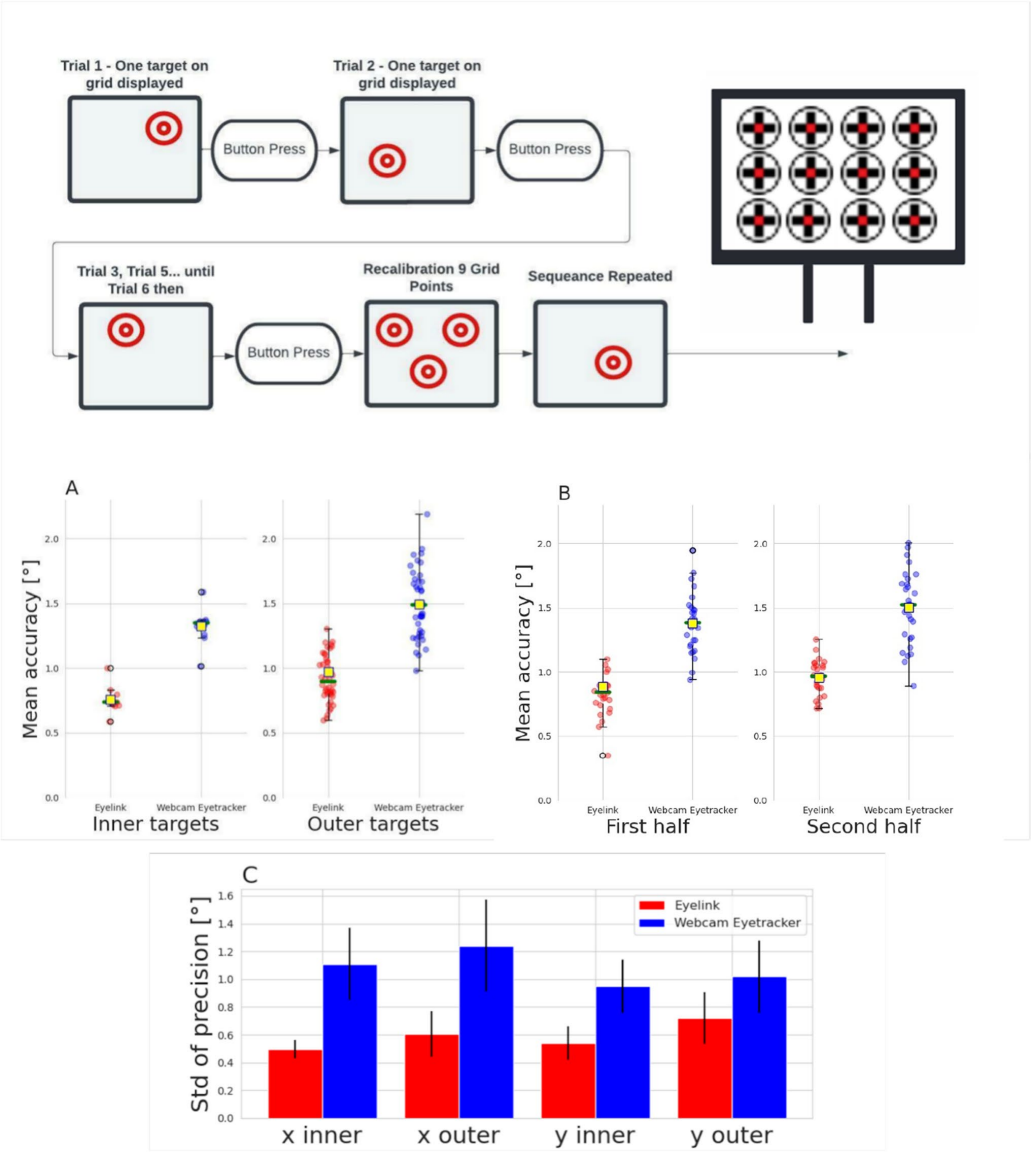
This figure represents the outcomes of the Large Grid task and includes the following comparisons: **A** Spatial accuracy: distance of the detected gaze to the target, comparing EyeLink and webcam-based eye trackers and inner versus outer eccentricity targets; **B** Spatial accuracy with first vs. second half of the task: comparing EyeLink and webcam-based eye trackers and the first versus the second half of the task; **C** Precision: comparing EyeLink and webcam-based eye trackers, horizontal (*X*) versus vertical (*Y*), and inner versus outer eccentricity targets. The *error bars* represent the standard deviation of the precision measurement.

To determine spatial accuracy, we calculated the distance between the average fixation-centroid and target in visual angle in the grid task for each location and subject and then calculated the mean (M) and standard deviation (SD) across subjects. Using this approach, we found that the average accuracy for the webcam-based system was M = 1.45°, SD = 0.76°, and the average accuracy for EyeLink was M = 0.91°, SD = 0.54°. To explore variances in accuracy in more depth, we also investigated how accuracy differed based on the eccentricity of the targets (inner vs. outer points of the screen). Further, we investigated whether the participants' performance remained consistent over time. To achieve this, we compared the precision and accuracy of participants in the first and second halves of the task. This approach allowed us to determine if there were any significant effects of temporal decay on the quality of their performance (Fig. 6). Accordingly, we conducted a three-way ANOVA (Table 2), with the factors eye-tracker type (webcam-based vs. EyeLink), spatial eccentricity (inner vs. outer targets), and first vs. second half of the task. The factor eye tracker type revealed a significant difference (*p* < 0.001, *F* = 117.5), indicating that EyeLink did have better accuracy than the webcam-based approach. Also, the factor spatial eccentricity was significant (*p* < 0.01, *F* = 10.416), showing that inner targets (webcam-based: 1.35°, EyeLink 0.85°) had better accuracy than outer targets (webcam-based: 1.50°, EyeLink 0.95°). The factor first vs. second half of the task was non-significant (*p* = 0.075), i.e., we have no evidence that the accuracy changed systematically throughout the task. Also, none of the interactions revealed a significant difference.

**Table 2:** Accuracy: ANOVA results of the mean distance to target between different subsets division. *F*: test statistic for hypothesis testing; p-unc: uncorrected *p* value for determining statistical significance; η2: effect size measure indicating the proportion of variance explained by the independent variable; *df*: degrees of freedom representing the number of independent observations used to estimate the variance in the data

Mean subsets division	Sum of squares	*df*	Mean square	*F*	p-unc	η2
First vs. second half of the task	4577.853	1.0	4577.853	3.157	.075	.002
Spatial division	15100.74	1.0	15100.740	10.416	.001	.008
Eye-tracker type	170455.2	1.0	170455.194	117.583	< .00001	.087
First vs. second half of the task * spatial division	428.502	1.0	428.502	.295	.586	.0002
First vs. second half of the task * eye-tracker type	110.956	1.0	110.955	.076	.782	.00006
Spatial division * eye-tracker type	284.027	1.0	284.027	.195	.658	.0001
First vs. second half of the task * spatial division * eye-tracker type	213.669	1.0	213.669	.147	.701	.0001
Residual	1777271	1226.0	1449.650	NaN	NaN	NaN

Next, we aimed to test for spatial precision, which is a measure of variance. For eye-tracking systems, precision refers to the spread in the systems’ prediction given a subject repeatedly looks at the same location, i.e., the input is the same. However, as our fixation-grid task did not include repeated fixations on the same target location for the same subject, we could not perform a within-subject precision calculation. Instead, we took data from different subjects but at the same grid-fixation location. Additionally, we investigated RMS-S2S (root mean square of successive displacements) which is used in eye-tracking systems to evaluate the precision of gaze position signals within a measurement (subject). It measures the average displacement magnitude between consecutive gaze positions, serving as an indicator of the smallest discernible eye movement amidst background noise (Niehorster et al., 2020a, b; Holmqvist & Blignaut, 2020). This was done separately for the horizontal and vertical components of the eye movements. The results for the webcam-based system were *X*-axis M = 1.66° and SD = 0.41°, for *Y*-axis M = 1.42° and SD = 0.45°. For EyeLink the results were *X*-axis M = 0.79°, SD = 0.23° and *Y*-axis M = 1.01° and SD = 0.31°. Please note that the accuracy of this metric relies on a stable sampling rate, which may be challenging to achieve with a webcam-based system. Because of this, we also investigate the grand mean of SD not within but across the subjects. For the webcam-based system was *X*-axis: M = 1.22°, SD = 0.32°, *Y*-axis: M = 1.00°, SD = 0.25°. For EyeLink, the *X*-axis was: M = 0.58°, SD = 0.15°, *Y*-axis: M = 0.68°, SD = 0.19° (Fig. 6C). We did expect precision values to be higher, i.e., worse, for both eye-tracking systems compared to the results from Ehinger et al. (2019) and other reports based on within-subject calculations. Therefore, the relative difference between the tested eye-tracking systems and not the absolute numbers is the most important to consider here. Accordingly, we calculated two separate ANOVAs for *X* and *Y* coordinates as dependent variables and the factors eye-tracker type and spatial eccentricity as independent factors (Tables 3 and 4). The results revealed that precision values were significantly different between both eye trackers for both *X* (*p* < 0.001, *F* = 183.940) and *Y* dimensions (*p* < 0.001, *F* = 63.594), with the EyeLink system demonstrating lower (better) precision values. Spatial eccentricity was also significantly different for both *X* and *Y*, demonstrating that inner targets generally had higher precision than outer targets. Remarkably, the interaction between the two factors was non-significant. That is, the dependence of precision on eccentricity is not significantly different between the two eye trackers. In conclusion, precision values for EyeLink were only about 50% better than for the webcam-based system, and a central target location improved precision for both systems similarly.

**Table 3:** Precision: ANOVA results of standard deviation around target across participants for *X* coordinates related fixations. *F*: test statistic for hypothesis testing p-unc: uncorrected *p* value for determining statistical significance; η2: effect size measure indicating the proportion of variance explained by the independent variable; *df*: degrees of freedom representing the number of independent observations used to estimate the variance in the data

Mean subsets division	Sum of squares	*df*	Mean square	*F*	p-unc	η2
Eyetracker type	11.246	1.0	11.246	183.940	**< .00001**	.630
Spatial division	.271	1.0	.271	4.446	**.037**	.039
Eye tracker type * spatial division	.271	1.0	.002	.0464	.829	.0004
Residual	6.603	108.0	.061	NaN	NaN	NaN

Bold text highlight the significance of the observation

**Table 4:** Precision: ANOVA results of standard deviation around target across participants for *Y* coordinates related fixations. *F*: test statistic for hypothesis testing p-unc: uncorrected *p* value for determining statistical significance; η2: effect size measure indicating the proportion of variance explained by the independent variable; *df*: degrees of freedom representing the number of independent observations used to estimate the variance in the data

Mean subsets division	Sum of squares	*df*	Mean square	*F*	p-unc	η2
Eyetracker type	2.901	1.0	2.901	63.594	**< .00001**	.370
Spatial division	.289	1.0	.289	6.341	**.013**	.055
Eye tracker type * spatial division	.057	1.0	.057	1.268	.262	.011
Residual	4.928	108.0	.045	NaN	NaN	NaN

Bold text highlight the significance of the observation

### Smooth Pursuit

**Fig. 7:**
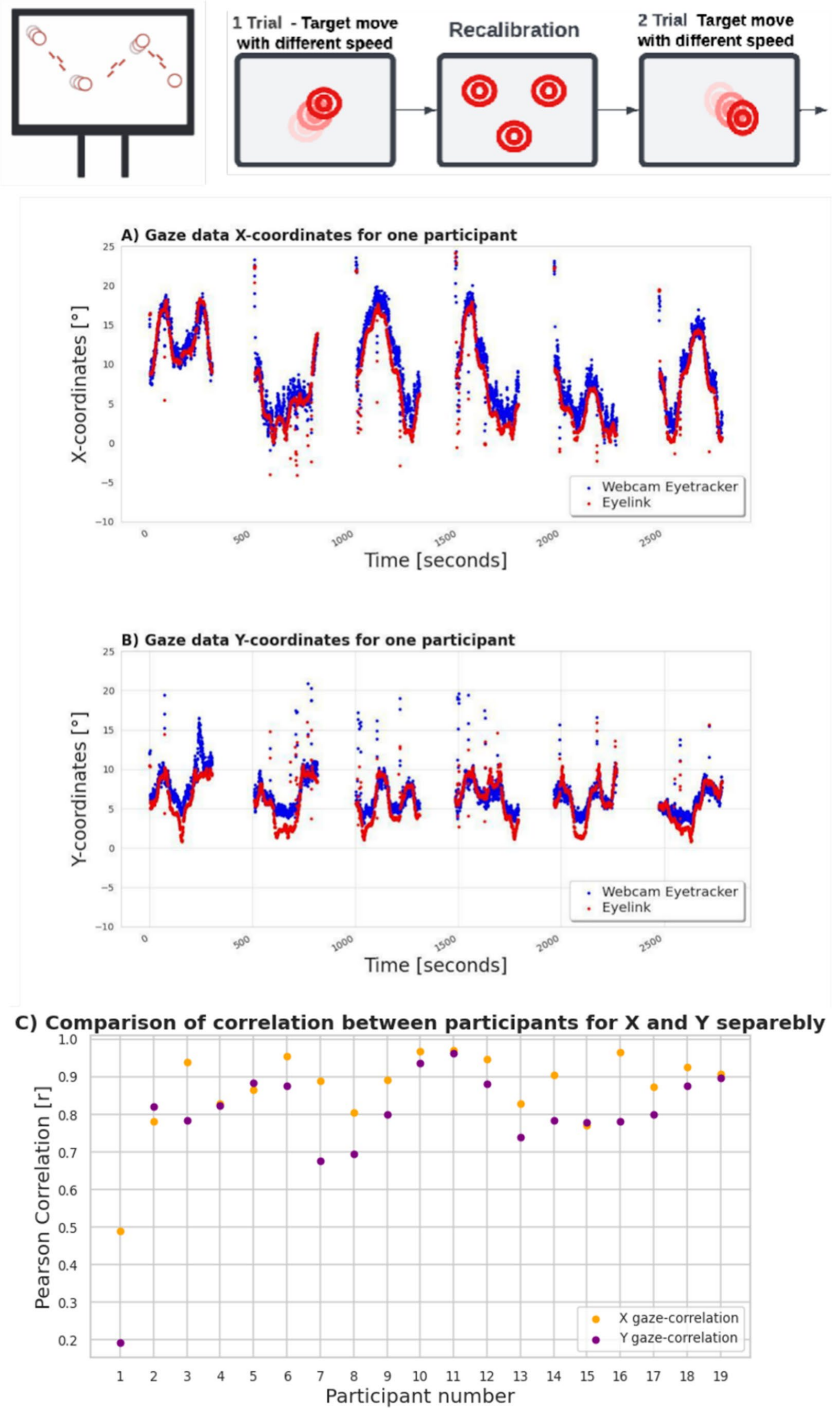
The panel represents scatter plots **A** and **B** with one participant's raw gaze data, showing the gaze data's alignment during the Smooth Pursuit task, separably for the *X*- and *Y*- axes. **C** The gaze data correlation for each participant separately. We could observe the low performance of one participant; however, the rest of the results remain similar

To analyze the Smooth Pursuit task, we aimed to look at the overall correlation of the unprocessed gaze signal between the two eye-tracking systems. We chose this method because it is independent of the fixation detection algorithm and we do not have ground truth available, i.e., where the participants look at any moment in time. Thus, it complements the fixation-based analysis performed in the grid task. We reduced biases and possible deficiencies of that newly introduced algorithm. Accordingly, we first removed all data points from both eye trackers where the EyeLink system reported data loss or where webcam-based confidence was zero. Then, we calculated for each subject a Pearson correlation separately for *X* and *Y* for both eye trackers. In the final step, we averaged these values across subjects. This analysis revealed a strong positive and significant correlation between the webcam-based system and the EyeLink system (Fig. 7A, B). For the horizontal component, the average correlation coefficient across subjects was *r* = 0.86 (*p* < 0.01), and for the vertical component, it was *r* = 0.78 (*p* < 0.01). Such a strong correlation between EyeLink and the webcam-based system underlines the good raw data quality of the webcam-based approach during periods of continuous eye movements. Analyzing the individual correlation between subjects, we observe that this high correlation is robust and consistent for almost all subjects (Fig. 7C).

### Free View

**Fig. 8:**
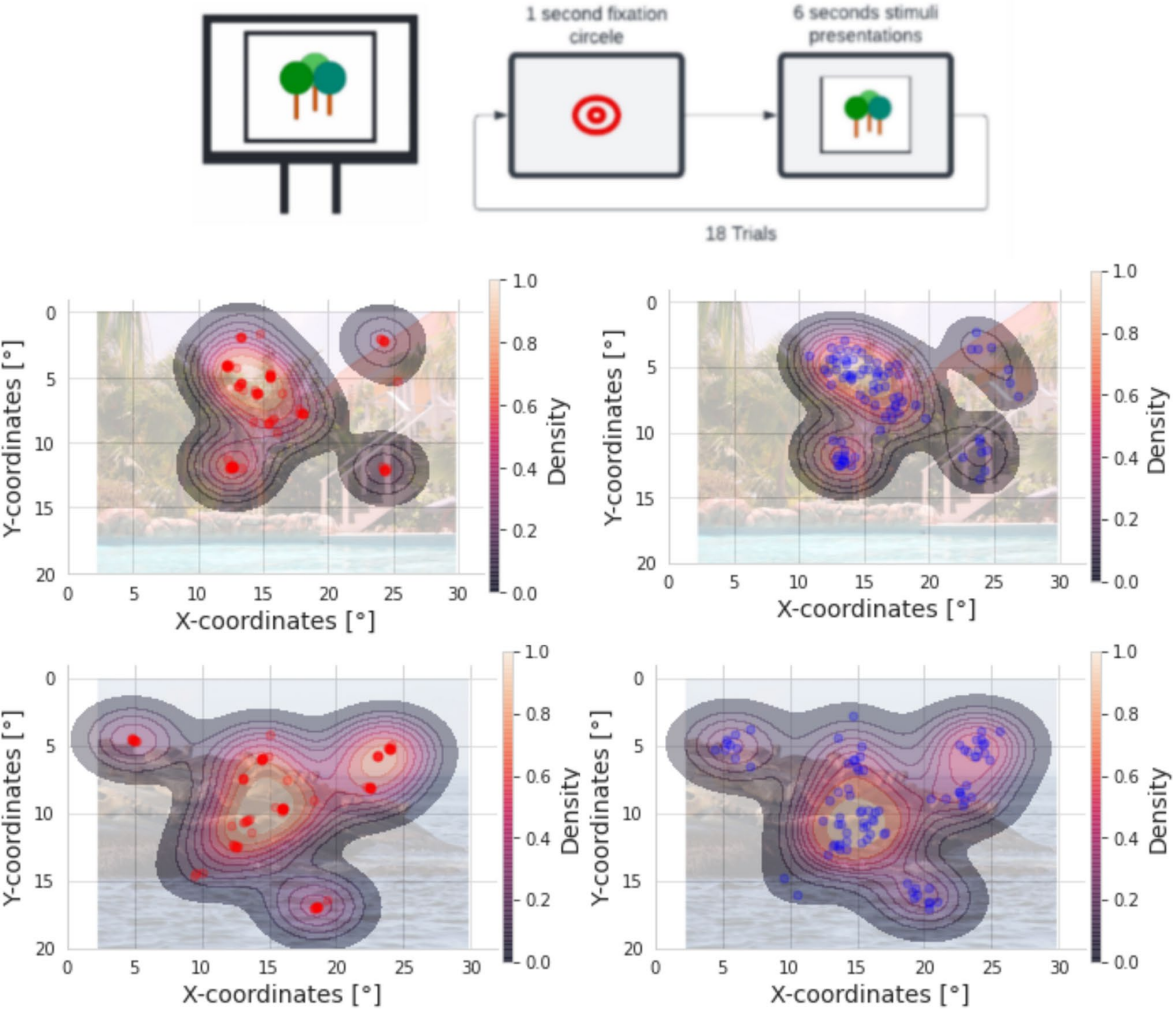
Heatmaps with two scatter plots from the participants and two different trials, where *left-red* represents EyeLink and *right-blue* webcam-based eye tracker. The corresponding stimuli image is used as the backdrop for the heatmap in each task. The values from 0 to 1 in the color bar represent the normalized density values of the kernel density estimate. From the given graphics, we could conclude that the gaze data are assigned roughly to the same objects in the image; however, there is a higher variance across the webcam gaze data distribution

We analyzed the gaze patterns during Free View to compare the two eye-tracking systems in a more natural task. A qualitative visual comparison of single-subject gaze data showed a great overlap between the two eye-tracking systems (Fig. 8). To verify these qualitative observations, we then performed a Pearson correlation of the raw gaze signals in the same way we did in the smooth pursuit task. The outcome showed a strong correlation between the raw gaze signals between the two eye-tracking systems. For coordinates *X* (*r* = 0.83, *p* < 0.01) and *Y* (*r* = 0.84, *p* < 0.01). These results confirmed the data quality of the newly introduced webcam-based system is also during a Free View scenario.

### Head Movements

**Fig. 9:**
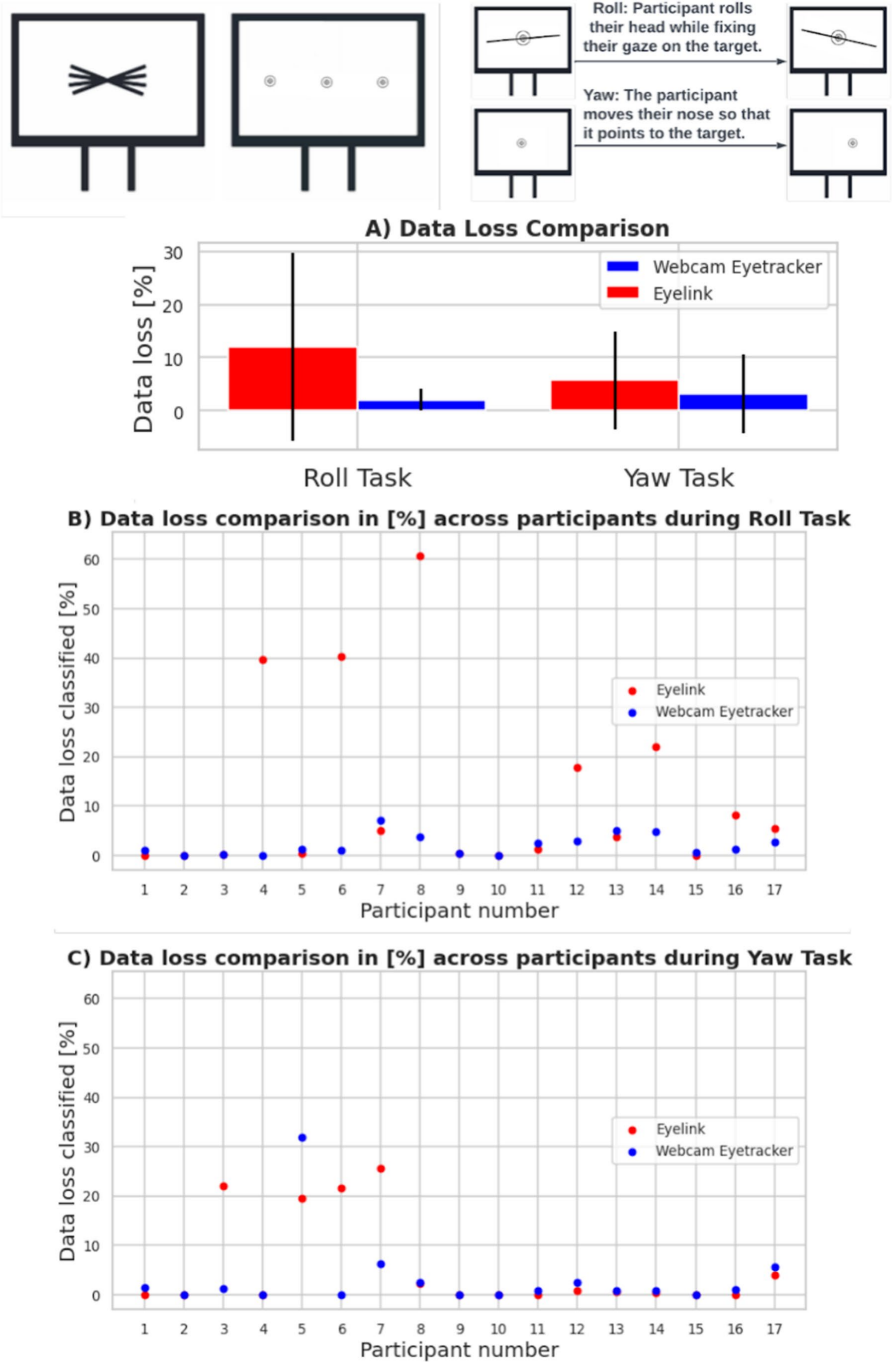
Percentage representation of overall data loss for Yaw and Roll tasks (Head Movements tasks; **A**). This analysis shows consistent small data loss over many subjects. However, we could also see the high data loss over a few participants (**B** and **C**) from the EyeLink system and high data loss for one participant from the webcam-based eye tracker

We compared the amount of data loss between two eye trackers for the two tasks that included Head Movements. On average, data loss was lower for the webcam-based system compared to EyeLink for both tasks: In Fig. 9A, the roll movements task results for the webcam-based eye tracker showed a mean (M) of 2.05% with a standard deviation (SD) of 2.05%, while the results for the EyeLink had a mean of 12.09% with a SD of 17.74%. Similarly, the yaw movements task for the webcam-based eye tracker had a mean of 3.24% with an SD of 7.41%, and for the EyeLink, the mean was 5.70% with an SD of 9.26%. We performed paired wise *t* test to compare data loss from the Roll task (*t* = – 2.28; *p* = 0.03), which shows a significant difference between the two eye-tracking systems; however, the results for the Yaw task (*t* = – 1.11; *p* = 0.28) were not significant. These results show that the webcam-based eye tracker was more robust to roll head movements than the EyeLink 1000. The EyeLink system identifies instances of data loss and categorizes them as eye blinks, with the start and end of the blink event being marked. Consequently, it is difficult to ascertain whether data loss or blink has been detected by this system accurately without additional data processing. On the other hand, the webcam eye tracker records data continuously, with data loss being represented as unreliable, as indicated by the low confidence levels that are usually 0.0. Table 4 provides comprehensive information on data loss and other relevant metrics.

### Results summary

Table 5 provides a comprehensive summary of all measurements obtained in this study. It is noteworthy that accuracy and precision measurements were exclusively available for the Large Grid task, and no offset-related fixations were observed, as described in the Large Grid section. We reported a total number of fixations without pre-processing, with a native sampling rate for the system as well as offset-related fixation which were pre-processed and downsampled. Insufficient data due to head rolls can impede the ability to accurately perform data interpolation and downsampling, resulting in a lack of correlation analysis for head roll tasks.

**Table 5:** Comprehensive summary of all metrics used in this paper and detailed information about each eye-tracking system with different tasks

Task name	Metrics	Large Grid	Smooth Pursuit	Free View	Yaw	Roll
Webcam-based	Mean gaze samples loss in %	2.63	3.06	1.54	3.24	12.09
	Standard deviation gaze samples loss in %	1.96	2.72	1.29	7.41	17.74
EyeLink	Mean gaze samples loss in %	2.08	7.24	2.39	5.7	2.05
	Standard deviation gaze samples loss in %	2.14	19.4	1.42	9.26	2.05
Webcam-based and EyeLink	Mean correlation *X* coordinates	0.93	0.78	0.83	-	-
Webcam-based and EyeLink	Mean correlation *Y* coordinates	0.89	0.86	0.84	-	-
Webcam-based	Total number of fixations	9278	8943	2950	-	-
EyeLink	Total number of fixations	17640	6382	5256	-	-
Webcam-based	Total number of offset-related fixations	619	-	-	-	-
EyeLink	Total number of offset-related fixations	615	-	-	-	-
Webcam-based	Mean accuracy [°]	1.45	-	-	-	-
EyeLink	Mean accuracy [°]	0.91	-	-	-	-
Webcam-based	Standard deviation accuracy [°]	0.76	-	-	-	-
EyeLink	Standard deviation accuracy [°]	0.54	-	-	-	-
Webcam-based	Mean precision [°]	1.23	-	-	-	-
EyeLink	Mean precision [°]	1.02	-	-	-	-
Webcam-based	Standard deviation precision [°]	0.33	-	-	-	-
EyeLink	Standard deviation precision [°]	0.26	-	-	-	-

## Discussion

Here, we compared a new webcam-based eye tracking algorithm to the well-established EyeLink system using a standardized test battery (Ehinger et al., 2019). While EyeLink showed, as expected, better accuracy and precision for fixation-based analysis than the webcam-based approach, these differences were only about 0.5° in size. In turn, this marks a huge improvement of the webcam-based method in accuracy and precision of about 300% compared to earlier webcam-based approaches (Papoutsaki et al., 2017). With this improvement, our approach comes close to the accuracies reported for mobile eye-tracking devices (Ehinger et al., 2019). Further, the raw gaze signal showed a consistent, robust correlation between the two eye-tracking systems. Finally, a surprising but encouraging finding was that the difference of webcam-based systems in data loss differed from the EyeLink in Roll movement task, showing that EyeLink was more susceptible to vertical head movements than the webcam-based solution.

### Limitations and possible improvements

While our results demonstrate a substantial improvement in webcam-based eye tracking, several improvements can still be made, and a few limitations are worth mentioning.

First, we used in the analysis of several tasks not accuracy and precision, but correlation as the relevant metric. This decision was motivated by the lack of ground truth in these tasks. In the Large Grid task, the participants are explicitly instructed to fixate on a specific location. Any deviation of gaze from that location results in a finite accuracy. In construct, in the other tasks, the subjects might variably explore the visual stimulus (e.g., in the Free View task) and, as a consequence, no deviation from a ground truth can be computed. Calculating the cross-correlation of the data obtained by the two eye trackers gives an objective and unbiased metric. Further, it is independent of any event detection algorithm manipulation. Nevertheless, we acknowledge that correlation, as a metric, does not cater to constant offsets and constant scaling. In other words, even when there are considerable disparities in accuracy and precision, signals could still display a high degree of correlation. To address this inherent limitation of correlation, we examined these aspects within the Large Grid task (mean correlation 0.93, Table 5), which specifically focuses on investigating accuracy and precision. By identifying and analyzing any constant offsets and scaling within this task, we could more effectively utilize correlation as a useful measure in our other tasks. In other words, when interpreting the correlation values, we have to bear in mind that a limited accuracy and precision as observed in the Large Grid task presumably applies to the other tasks as well.

We also derived the motivation for choosing this metric from the prior application of this metric in a comparative study between humans and monkeys to unveil both convergent and divergent patterns of movie viewing (Shepherd et al., 2010). Furthermore, the correlation between gaze data and behavioral studies is commonly employed in the exploration of decision-making processes (Fletcher & Zelinsky, 2009; Weill-Tessier & Gellersen, 2018).

The second limitation of any current webcam-based eye tracking is the comparably low sampling rate matching standard video frame rates of on average approximately 30 Hz. There are many other vendors using low-sampling-rate devices, for example Tobii Sticky (https://www.tobii.com/products/software/online-marketing-research/sticky), RealEye.io (https://www.realeye.io/), and GazeRecorder (https://gazerecorder.com/). As of today, webcam-based eye-tracking systems primarily work with gaze data sampled at 30 Hz because most webcams are working at such frequencies. And while the availability of these systems does not directly suggest their correctness or usefulness, it indicates that some (UI/UX/market/psychology) researchers derive meaningful value from these 30-Hz systems. Hence, webcam-based eye tracking is not yet suitable for detecting fast saccadic events (Aljaafreh et al., 2020; Semmelmann & Weigelt, 2018), microsaccades (Martinez-Conde et al., 2009), or other signals that are only detectable on very transient timescales. Interestingly, it should be noted that at the moment of writing this paper, the webcam-based system is already adapted and ready for webcams with higher fps rates, so once these are more widely available, the system will also be able to handle more transient events. We believe that in the near to mid-term future, such sampling rates are also consistently achievable in online webcam-based eye-tracking studies. Third, a promising approach seems to be using the EyeLink gaze and fixation data as ground truth and optimizing fixation detection for the webcam-based gaze data by machine learning algorithms. The dispersion-based fixation detection algorithm currently in use is a custom-built version that is openly available at (https://github.com/Labvanced/eye tracking-fixation-detection). It is important to acknowledge the existence of alternative offline algorithms, such as the I2MC (identification by two-means clustering) algorithm (Hessels et al., 2017), for fixation detection in eye-tracking data. The I2MC algorithm is specifically designed to handle varying levels of noise and data loss, ensuring reliable and precise identification of fixations. However, this publication does not include offline analysis, as offline algorithms may encounter different challenges and serve different applications and use cases.

Fourth, one might consider the implications of the virtual chinrest, which is a unique and novel approach to online testing used to reduce head movements. The virtual chinrest is implemented by pausing the trial as soon as the participants’ head moves out of the allowed range of the chinrest and showing the participant their face with the masked overlay. Only after bringing their head back into position does the trial continue. According to our observations, the vast majority of participants learned rather quickly that they were not allowed to move their heads and kept very still. However, some participants might have had more difficulties with this than others, so the number of interruptions and pauses within a trial might have been large for some subjects. Also, the implications of pausing and resuming a trial are arguably different depending on the paradigm. Such a virtual chinrest might be problematic for some paradigms where uninterrupted stimuli presentation is a prerequisite. One possible solution could be to discard such trials and only analyze trials without interruptions.

The fifth area for potential improvement pertains to calibration, which is a critical component of the EyeLink system that involves both calibration and validation tasks. Our calibration protocol involved presenting nine fixation targets twice, which theoretically is a straightforward and expeditious process. However, in practice, several other parameters, such as the subject's distance, the focal point of the lens, and the pupil size, among others, can impact the accuracy of the calibration. Furthermore, the calibration procedure had to be set up by the researcher, who then had to verify its quality. Consequently, in many cases, the entire calibration process had to be repeated to achieve the desired low calibration error. The duration of the EyeLink calibration process varied from 2 to 5 min. While the webcam-based eye tracker calibration time is adjustable in the algorithm, we chose the 5-min calibration (~ 120 points) for the current study. This is rather long for an eye-tracking calibration and requires the subject's close attention during the whole period. However, we can also clearly see that the shorter the calibration, the less accurate the results. This is because the neural network used for pose estimation will generalize better the more customized training samples it can use. We even tested an ~ 8-min calibration but considered it impractical after the initial online tests of some subjects. Hence, for most of our preliminary online and in-lab data recordings, we used the 5-min calibration. We see an initial success rate of about 80% for the calibration, which seems acceptable. However, while such a calibration time seems to work for dedicated subjects using crowdsourcing platforms, it seems that it will be necessary to shorten further and simplify the calibration to expand webcam-based eye tracking to other use cases. The duration of the re-calibration time can be customized to suit the needs of the specific experiment and it comprises seven equally spaced fixation targets that are presented for approximately 15 s. For the Large Grid experiment, the entire re-calibration process took about 2 min, while for the Smooth Pursuit experiment, it took 1.5 min and for the Free View experiment, it took only 0.60 ms. As a result, approximately four extra minutes were added to the total experiment time. Notably, the Head Movements task did not require a re-calibration procedure since the entire experiment, including subject preparation and calibration, lasted about an hour. Therefore, an additional 4 min for recalibration should not pose any challenges, even for large-scale studies. However, as previously mentioned, the recalibration procedure can be completely deactivated or adjusted.

Finally, it is important to note that we here focused on and reported the accuracy of webcam-based eye tracking on a desktop, as we believe this has the most significant impact on professional empirical research at the moment. When applying the same method for mobile devices, the overall accuracy will likely be better, as the calibration is performed on a much smaller area. However, given the much smaller screen area and additional degrees of freedom due to arm and hand movements, we consider smartphone eye tracking experiments still experimental and plan to investigate it more closely in the future.

### Real-time and client-side vs. post hoc gaze estimation

The webcam-based eye-tracking system we are proposing here performs real-time gaze estimation directly on the participant's device. An alternate solution would be to record and save a webcam video while the participant is doing the task and perform the gaze estimation post hoc without any real-time restrictions. In fact, this is what several other frameworks and researchers have been doing before (Saxena et al., 2022; Schultz et al., 2007; Desai et al., 2019). While this distinction might sound like a smaller technicality, it has large implications that can be compared in the domains of accuracy, utility, and data privacy.

Regarding accuracy, webcam-based real-time gaze estimation is limited to the capabilities of the participant's device. In contrast, for post hoc estimation, a researcher can use the most powerful machine available. This can be considered a clear advantage for post hoc processing because CPU and especially GPU hardware specifications are crucial in machine learning architectures. Hence, to speed up the performance of the real-time system, we employed the latest web technologies such as web workers, i.e., parallel processing techniques, and fast inference methods based on web assembly and Tensorflow (Reiser & Bläser, 2017; Smilkov et al., 2019). Furthermore, the present system allows us to check the performance of the participants’ devices, specifically CPU and GPU speeds, and reject setups below an adjustable threshold beforehand. These measures mitigate hardware-based deficiencies of real-time inference to a considerable degree but also reduce the number of eligible remote subjects. However, given the almost unlimited number of subjects one can reach with online crowdsourcing techniques (Chittilappilly et al., 2016), this seems to be an appropriate trade-off. Also, the accuracy is only partly dependent on the performance of the inference machine; most important is, of course, the data quality. Using a real-time inference mechanism, we can create precise millisecond timestamps for each processed webcam image because we can directly access and analyze the raw webcam stream. Instead, when creating an mp4/h264 encoded HTML video, there is data loss both on the video content due to image compression, as well as on the timestamps because the video does not create millisecond precise timestamps for each image and because the frame rate is not necessarily 100% constant across time. This data loss and temporal imprecision could be circumvented by not saving a compressed video but instead single uncompressed images. In this case, however, the amount of data to handle would be enormous, up to 180 MB/s of data per subject for an HD webcam. A final important consideration is that our real-time gaze estimation enables us to implement a virtual chinrest, effectively decreasing the number of head movements during the recording to a controllable degree. Without a real-time calculation of the head pose, this is, of course, not possible, and hence post hoc inference has to deal with a much larger amount of head movements. Our intuition is that the virtual chinrest, and consequently reduced head movements, have a strong positive impact on the reported accuracy of our system. This idea is supported by the results of a recent study from Saxena et al. (2022), who conducted a webcam-based eye-tracking study but instead used a post hoc inference strategy and reported an average accuracy of about 2.58° – less accurate compared to what we report with an enabled virtual chin-rest. However, future research directly comparing the accuracy with and without virtual chinrest will bring more conclusive evidence.

A second important factor regarding real-time vs. post hoc gaze estimation is the question of utility, meaning which experiments are possible to realize depending on the method chosen. Most importantly, real-time gaze estimation can use the predicted gaze data and even online calculated fixations to interact with the experiment. For instance, using our system, one can force the subject to look at a fixation cross before showing a specific stimulus, or a subject can choose by fixating on an object for a certain amount of time. One could even stream the gaze location between participants in a multi-participant study or invent other more advanced use cases. As post hoc inference, one does not have any of these capabilities, it is clear that real-time analysis possesses a much higher utility.

A final consideration is the aspect of data privacy. According to Bozkir et al. (2023), eye movements could be associated with various attributes such as gender, sexual preferences, body mass index, health status, etc. The collection of eye-tracking data raises concerns regarding user privacy, as it contains unique characteristics that can be exploited for example for targeted advertising purposes. The studies mentioned in the text primarily focus on high-sampling-rate devices in virtual reality (VR) and behavioral authentication. Mentioned methods exhibit limited accuracy and require the incorporation of more intricate fine-grained features to effectively differentiate and identify personal attributes. In contrast, the authors highlight the potential of iris-based authentication, which offers high accuracy in privacy identification by capturing iris-texture data. Identification methods explained in the paper (Bozkir et al., 2023) rely heavily on high sampling rates in combination with behavioral features or iris images. It should be noted that the webcam-based eye-tracker system discussed in the paper does not store raw image data. However, the authors of this publication demonstrate a strong awareness of the potential risks associated with technology development. They emphasize the importance of exercising caution and considering the implications of privacy and security while advancing new technologies.

In the present work, we argue for real-time gaze estimation on the participants' devices. This means that no face/image data ever leaves the participants’ devices, but only the predicted eye location is sent to the server. Doing post hoc inference requires that the video data of the subjects’ faces must be sent from the participants’ devices over the Internet to a server controlled by the researcher. One could decide to only scramble/blur the face data or only extract the eye images client side, save those extracted or modified images on a server, and post-process these images to arrive at gaze data. However, eye-only images (even if harder to use for identification) are still private data and the amount of data processing needed for this on the participant device is still significant. Hence, the additional step to also do the gaze prediction in real time, on the participant's devices, and only store gaze information seems like the most elegant and privacy-preserving solution. Even if this data would be client-side (end-to-end) encrypted and only decrypted for data analysis, this would still expose the raw face data to the system of the person analyzing the data. To our understanding, this not only constitutes a relatively large risk of a data privacy breach, e.g., by lack of secure data transport, storage, or access mechanisms but also stands against the idea of modern data privacy laws such as GDPR or HIPPA (Tovino, 2016). In our opinion, this critical fact alone strongly argues in favor of local real-time gaze inferences. In summary, our preferences and judgments are rather clear.

With state-of-the-art technologies, real-time gaze estimation can be considered more accurate, feature complete, and especially secure and privacy-preserving compared to post hoc inference methods. Potential accuracy deficiencies due to inferior hardware can be mitigated using modern web technologies and should finally be overcome by better computing devices.

### Outlook

The use cases for accurate, cheap, and real-time eye tracking, working natively in the web browser without needing dedicated hardware, are enormous. The potential benefits range from improved marketing approaches (Białowąs & Szyszka, 2019), more interactive gaming (Alhargan et al., 2019), improved videoconferencing tooling (Adams, 2020), to more insightful academic research (Orquin et al., 2019) and more accessible medical diagnostics and the treatment (Boraston & Blakemore, 2007; Guillon et al., 2014; Stuart et al., 2016; Bueno et al., 2019; Bek et al., 2020). We are glad to be part of this journey and hope our contribution will provide the next step toward this common goal.

## Footnotes

1 After clicking, there are two options: import and inspect, inspect works without having to create an account on the platform and allows a review of the logic of all the experiments used in this study. To review the Smooth Pursuit logic, go to the Task Editor tab and select the Smooth Pursuit task.

## Declarations

This research “Webcam eye tracking close to laboratory standards: Comparing a new webcam-based system and the EyeLink 1000.” was funded by the EU-project euSNN (MSCA-ITN-ETN H2020-860563).

Three of the four authors of this manuscript are employees of the Scicovery company, which is responsible for the development of the Labvanced platform and online-based webcam eye tracking.

The participants gave written consent, and the ethics committee approved the study of Osnabrück University (number 58/2021, issued on December 15th, 2021).

I hereby declare and give my consent for the publication of identifiable details, which can include graphs and source codes and/or details within the text (“Material”) to be published in the Journal Behavior Research Methods.

The code which was used for the fixation detection is publicly available under this link (https://github.com/Labvanced/eye-tracking-fixation-detection.). The data are publicly available as suggested by the editor repository Open Science Framework under this link (https://osf.io/cg3s7/?view_only=6e6657d18f5547fc9ce6fd1cc686ac47). The authors confirm that the data supporting the findings of this study are available within the article

The two sessions of the experiment are available online under these links:

• https://www.labvanced.com/player.html?id=28983

• https://www.labvanced.com/player.html?id=28969 (Head Movements)
